# Tubular cell senescence promotes maladaptive kidney repair and chronic kidney disease after cisplatin nephrotoxicity

**DOI:** 10.1172/jci.insight.166643

**Published:** 2023-04-24

**Authors:** Siyao Li, Man J. Livingston, Zhengwei Ma, Xiaoru Hu, Lu Wen, Han-Fei Ding, Daohong Zhou, Zheng Dong

**Affiliations:** 1 Department of Nephrology, The Second Xiangya Hospital of Central South University, Hunan Key Laboratory of Kidney Disease and Blood Purification, Changsha, China.; 2 Department of Cellular Biology and Anatomy, Medical College of Georgia at Augusta University, Augusta, Georgia, USA.; 3 Division of Molecular and Cellular Pathology, Department of Pathology, University of Alabama School of Medicine at Birmingham, Birmingham, Alabama, USA.; 4 Center for Innovative Drug Development and Department of Biochemistry and Structural Biology, University of Texas Health Science Center at San Antonio, San Antonio, Texas, USA.; 5 Research Department, Charlie Norwood VA Medical Center, Augusta, Georgia, USA.

**Keywords:** Nephrology, Cellular senescence, Chronic kidney disease

## Abstract

Cisplatin is a widely used chemotherapy drug; however, it induces both acute and chronic kidney diseases (CKD) in patients with cancer. The pathogenesis of cisplatin-induced CKD is unclear, and effective renoprotective approaches are not available. Here, we report that repeated low-dose cisplatin (RLDC) treatment of C57BL/6 mice induced chronic cellular senescence in kidney tubules, accompanied with tubular degeneration and profibrotic phenotype transformation that culminated in maladaptive repair and renal fibrosis. Suppression of tubular senescence by senolytic drugs ABT-263 and Fisetin attenuated renal fibrosis and improved tubular repair, as indicated by restoration of tubular regeneration and renal function. In vitro, RLDC also induced senescence in mouse proximal tubular (BUMPT) cells. ABT-263 eliminated senescent BUMPT cells following RLDC treatment, reversed the profibrotic phenotype of the cells, and increased their clonogenic activity. Moreover, ABT-263 alleviated the paracrine effect of RLDC-treated BUMPT cells on fibroblasts for fibrosis. Consistently, knockdown of *p16* suppressed post-RLDC senescence and fibrotic changes in BUMPT cells and alleviated their paracrine effects on renal fibroblast proliferation. These results indicate that persistent induction of tubular senescence plays an important role in promoting cisplatin-induced CKD. Targeting senescent tubular cells may be efficient for improvement of kidney repair and for the prevention and treatment of cisplatin-induced CKD.

## Graphical abstract

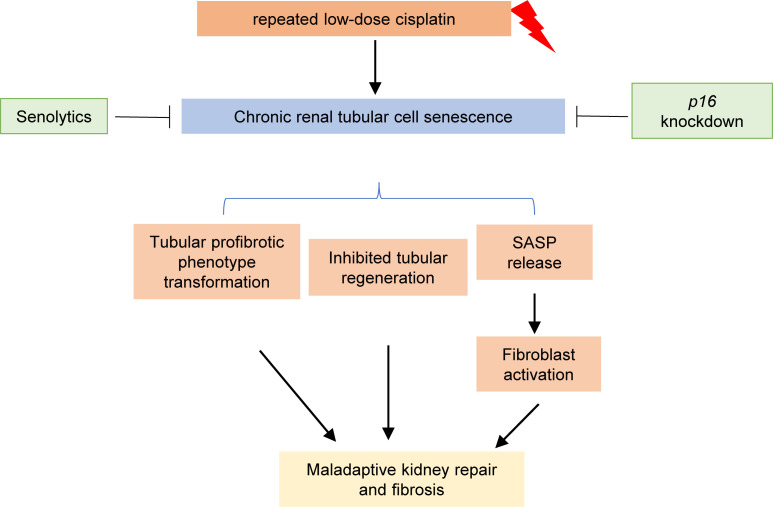

## Introduction

Cisplatin is a widely used chemotherapy drug for various solid tumors. However, it has notorious side effects in normal tissues, particularly in the kidney (1–3). Within days of initiating cisplatin chemotherapy, approximately 30% of patients develop acute kidney injury (AKI) that is characterized by rapid loss of renal function. In the long term, these patients may also experience a persistent decline in renal function within a few years after cisplatin treatment, suggesting the progression to chronic kidney disease (CKD) (4).

Tubular cell injury and death are the major pathological features of cisplatin-induced AKI (1–3). After acute injury, kidney tubules have the capacity to regenerate, which can lead to kidney repair (5). Depending on the severity and duration of the initial injury, kidney repair can be adaptive or maladaptive (5–9). Adaptive repair following mild injury may completely restore normal tubular structure and function. After severe or repeated episodes of AKI, maladaptive repair occurs, resulting in evolving tubular pathologies, chronic inflammation, capillary rarefaction, tubulointerstitial fibrosis, and glomerulosclerosis that contribute to gradual decline of renal function and progression to CKD (5–9). During maladaptive kidney repair, renal tubules are not only a victim of injury but also a key driver for disease progression (10). A subpopulation of injured tubules undergoes multiple alterations including dedifferentiation, cell-cycle arrest, cell senescence, autophagy, and metabolic changes. These responses are adaptive initially to overcome injury but become detrimental and promote CKD when sustained (5–10). In particular, these changes can cause injured renal tubules produce and release various profibrotic and proinflammatory factors to initiate a cascade of events leading to the pathological changes of CKD (5–10).

Cellular senescence is a status of permanent cell cycle arrest that progresses with age and can also be triggered by various stress such as DNA damage, oncogene activation, reactive metabolites, and others (11–13). Depending on the inducer and cell type, cellular senescence is regulated by mechanisms converging on pathways that eventually activate RB-P16 and/or P53-P21. Regardless of the loss of replicative capacity, senescent cells are apoptosis resistant and metabolically active, with characteristic morphologies and functions including a senescence-associated secretory phenotype (SASP), particularly. The SASP is a highly heterogeneous program that produces and secretes proinflammatory cytokines, chemokines, growth factors, and extracellular matrix (ECM) proteases (11–13). Acute senescence with restricted SASP has beneficial functions, whereas chronic senescence with persistent SASP contributes to age-related diseases, cancer, and other chronic diseases (13–15). Targeted elimination of chronic senescent cells by small-molecule senolytic drugs and/or disruption of the key events of senescence, such as SASP, by senomorphic agents have recently emerged as a promising therapeutic strategy for preventing or treating these diseases in both preclinical settings and clinical trials (13, 16, 17).

In the kidney, increased senescent cells are found in tubular cells and other cell types, including podocytes, mesangial cells, endothelial cells, and interstitial cells, under various disease conditions (18–23). Progressive cellular senescence is associated with tubular injury and kidney dysfunction in chronic renal ischemia induced by renal artery stenosis (24). Moreover, cellular senescence and the SASP are involved in many forms of CKD, such as diabetic kidney disease (22), obesity-related kidney disease (25), and IgA nephropathy (26). Despite these findings, the role of cellular senescence in cisplatin-induced kidney injury and repair is much less understood.

In this study, using repeated low-dose cisplatin (RLDC) treatment to model cisplatin-induced AKI to CKD transition, we demonstrate that RLDC induced a persistent cellular senescence both in murine renal tubules and in cultured mouse proximal tubular cells, leading to a profibrotic phenotype transformation for maladaptive repair and disease progression. Selective clearance of senescent tubular cells with senolytic drugs alleviated kidney fibrosis and improved tubular cell regeneration. Genetic inhibition of *p16* also suppressed RLDC-induced tubular cell senescence and profibrotic changes. These results suggest an important role of tubular senescence in determining the outcome of kidney repair after cisplatin-induced injury. Targeting senescent tubular cells may be an effective therapeutic approach to improve kidney repair for the prevention and treatment of cisplatin-induced CKD.

## Results

### Persistent renal tubular senescence is induced in post-RLDC mice and suppressed by senolytic drugs ABT-263 and Fisetin.

C57BL/6 mice were subjected to RLDC treatment consisting of 4 weekly injections of 8 mg/kg cisplatin (27). Compared with control mice, there was a 13-fold increase of *p16* mRNA in the kidneys of RLDC-treated mice, suggesting an induction of renal senescence (Supplemental Figure 1A, 4 w; supplemental material available online with this article; https://doi.org/10.1172/jci.insight.166643DS1). Notably, elevated *p16* was maintained at 4 weeks after the completion of RLDC (Supplemental Figure 1A, 8 w). Coimmunofluorescence staining of P16 and kidney injury molecule-1 (KIM-1), a marker for injured renal proximal tubules, further revealed that P16 was induced primarily in the nuclei of renal tubules positive for KIM-1 in post-RLDC mice, whereas it was not detected in control mice (Supplemental Figure 1B). The upregulation of *p16* was accompanied with the induction of both *p19* and *p21* mRNAs at 4 weeks after the completion of RLDC (Supplemental Figure 1, C and D). At this time point, multiple SASP factors, including connective tissue growth factor (*Ctgf*)*,* platelet-derived growth factor, B polypeptide (*Pdgfb*), *Tgfb*, *Il-6*, and *Tnfa*, were also induced in post-RLDC kidneys (Supplemental Figure 1, E–J), further suggesting a sustained induction of tubular cell senescence throughout the development of cisplatin-induced CKD.

**Figure 1:**
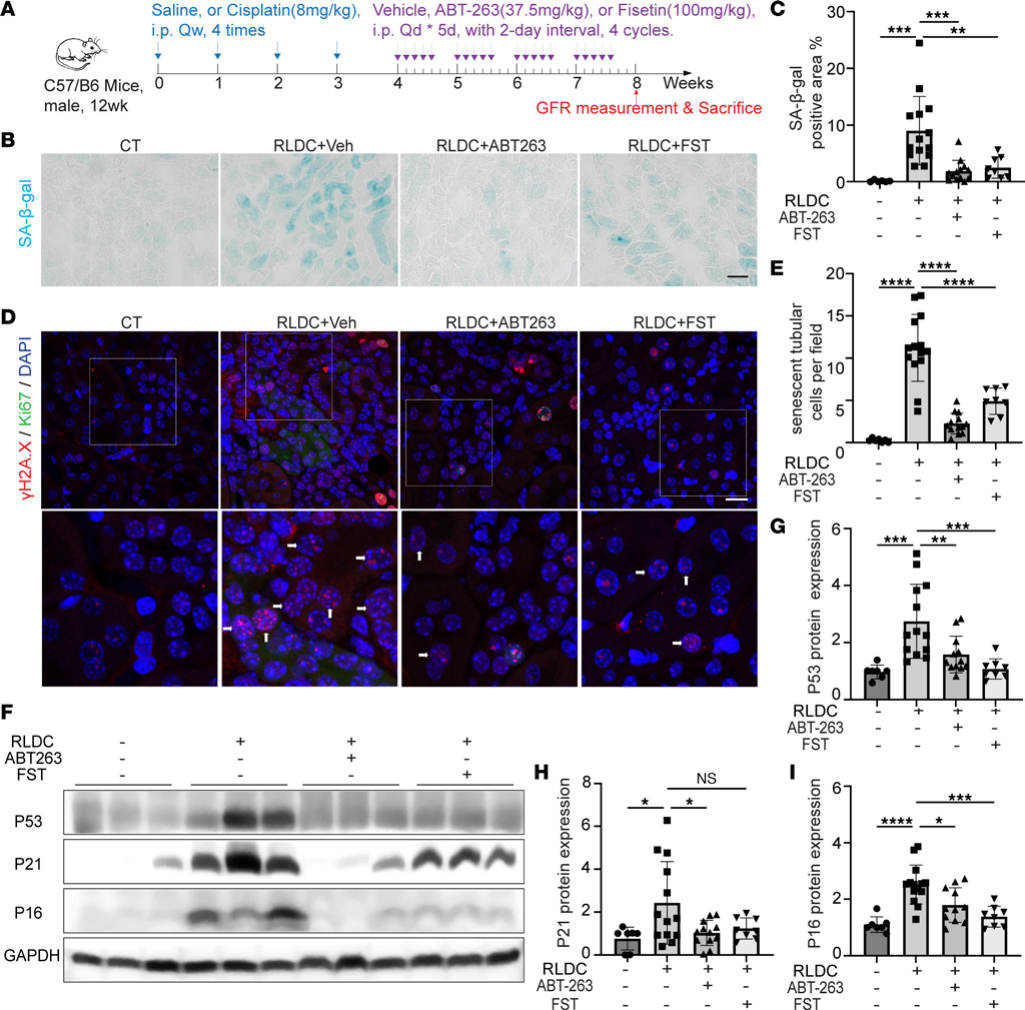
Persistent renal tubular senescence is induced in post-RLDC mice and suppressed by senolytic drugs ABT-263 and Fisetin. (**A**) Schematic diagram of treatment. C57BL/6 mice were subjected to RLDC treatment followed by 4-week treatment of ABT-263 (*n* = 13), Fisetin (FST) (*n* = 8), or vehicle (*n* = 14). Control (CT) mice (*n* = 6 or 7) were injected with saline. Kidneys were harvested at 8 weeks for analysis. (**B**) Representative images of SA-β-gal staining. Scale bar: 50 μm. (**C**) Quantification of SA-β-gal staining. (**D**) Representative images of costaining of γH2A.X (red), Ki67 (green) and DAPI (blue). Arrows indicate typical senescent tubular cells with 4 or more γH2A.X-positive foci and Ki67-negative. The bottom images are enlarged from the boxed areas in the top images. Original magnification, ×630 (original); ×1,260 (enlarged). Scale bar: 15 μm. (**E**) Quantification of γH2A.X-positive/Ki67-negative senescent tubular cells. (**F**) Immunoblot analysis of P53, P21, and P16. (**G–I**) Densitometry of P53, P21, and P16 expression. Quantitative data are presented as mean ± SD. One-way ANOVA with multiple comparisons was used for statistics. *P < 0.05, **P < 0.01, ***P < 0.005, and ****P < 0.001.

We then treated post-RLDC mice with 2 well-known senolytic agents, ABT-263 and Fisetin (FST), and verified their effects on tubular cell senescence. The dosage and treatment duration of these 2 drugs are outlined in Figure 1A. In addition to the increased expression of senescence-regulating genes and the SASP factors shown above, RLDC also induced the expression of senescence-associated galactosidase, β 1 (SA-β-gal), another commonly used biomarker of cellular senescence, in many renal tubules, which was largely inhibited by both ABT-263 and FST (Figure 1B). Quantitative analysis confirmed that the SA-β-gal positively stained areas were reduced from 9.01% in vehicle-treated mice to 1.88% by ABT-263 and to 2.52% by FST (Figure 1C). γH2A.X staining in Ki67-negative cells is another indication of senescence in maladaptive kidney repair (28, 29). In post-RLDC kidneys, we detected Ki67-negative tubular cells with 4 or more γH2A.X foci, the number of which was suppressed by ABT-263 and FST (Figure 1, D and E). Consistently, the upregulation of 3 senescence-regulating proteins, P53, P21, and P16, in post-RLDC kidneys was also significantly reduced by both senolytic drugs (Figure 1, F–I). These results confirm the inhibitory effects of ABT-263 and FST on renal tubular senescence in post-RLDC kidneys.

It is noteworthy that in control mice without cisplatin exposure and induction of tubular cell senescence, 4 cycles of ABT-263 or FST treatment did not show toxic effects in the kidney, as indicated by normal renal function and kidney histology (Supplemental Figure 2, A–D). In line with the rationale that the first-generation senolytic drugs such as ABT-263 and FST disable senescent cell antiapoptotic pathways (SCAPs) to cause apoptosis of senescent cells with a tissue-destructive SASP while nonsenescent cells remain viable (17), both ABT-263 and FST induced the expression of cleaved caspase-3 (C-CASP3), a marker of cell apoptosis, likely in senescent tubular cells in post-RLDC mice (Supplemental Figure 2E). On average, 6 C-CASP3–positive tubular cells per 400× field were induced by ABT-263 and 3 were induced by FST in post-RLDC mice (Supplemental Figure 2F). These results provide in vivo evidence that both senolytic drugs may selectively eliminate chronic senescent tubular cells by inducing apoptosis.

### ABT-263 and FST inhibit renal interstitial fibrosis in post-RLDC mice.

**Figure 2:**
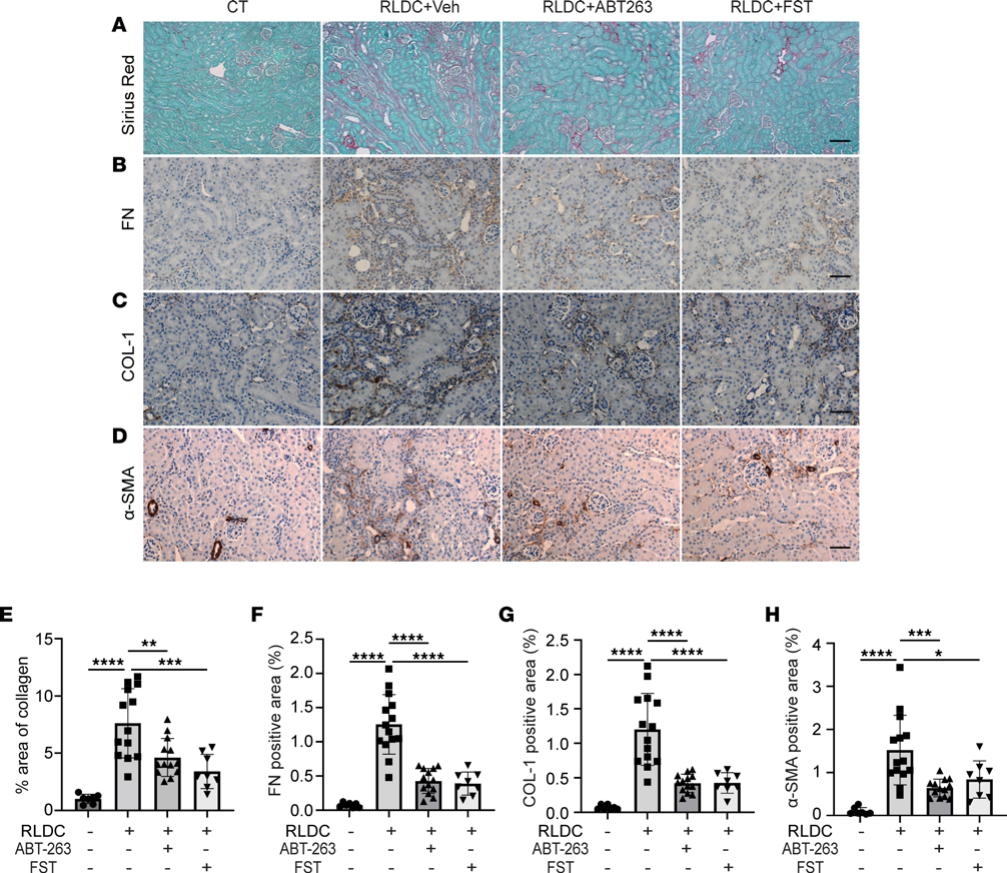
ABT-263 and FST inhibit renal interstitial fibrosis in post-RLDC mice. C57BL/6 mice were subjected to RLDC treatment followed by 4-week treatment of ABT-263 (*n* = 13), FST (*n* = 8), or vehicle (*n* = 14). Control (CT) mice (*n* = 7) were injected with saline. Kidneys were harvested at 8 weeks for histology and immunohistochemical staining. (**A**) Representative images of Sirius Red/Fast Green staining. Scale bar: 100 μm. (**B–D**) Representative images of FN, COL1, and α-SMA staining. Scale bar: 50 μm. (**E**) Quantification of Sirius Red/Fast Green staining. (**F–H**) Quantification of FN, COL1, and α-SMA staining. Positive signals from blood vessels and glomeruli were excluded. Quantitative data are presented as mean ±SD. One-way ANOVA with multiple comparisons was used for statistics. *P < 0.05, **P < 0.01, ***P < 0.005, and ****P < 0.001.

To determine the pathological role of renal tubular senescence in cisplatin-induced CKD, we first examined the effects of senolytics on interstitial fibrosis. Compared with control mice, RLDC induced a significant amount of collagen deposition in renal interstitium, as indicated by Sirius Red/Fast Green staining (Figure 2A). Both ABT-263 and FST suppressed fibrosis in post-RLDC mice, reducing the fibrotic areas from 7.61% in vehicle-treated mice to 4.62% and 3.40%, respectively (Figure 2, A and E). Consistently, immunohistochemical staining of fibronectin (FN) and collagen, type I (COL1) also showed increased accumulation of these ECM proteins in interstitial tissues surrounding the atrophic renal tubules in post-RLDC mice, which was again significantly attenuated by both senolytic drugs (Figure 2, B, C, F, and G). Moreover, RLDC promoted fibroblast activation, as indicated by the increased staining of actin α 2, smooth muscle, aorta (α-SMA) in renal interstitium, particularly around the atrophic tubules; this fibroblast activation was also ameliorated in post-RLDC mice treated with ABT-263 and FST (Figure 2, D and H). Together, these results suggest a profibrotic role of sustained renal tubular senescence in post-RLDC mice. Reducing tubular senescent cell burden by senolytic drugs alleviates RLDC-induced renal interstitial fibrosis.

### ABT-263 and FST improve renal function and tubular repair in post-RLDC mice.

**Figure 3:**
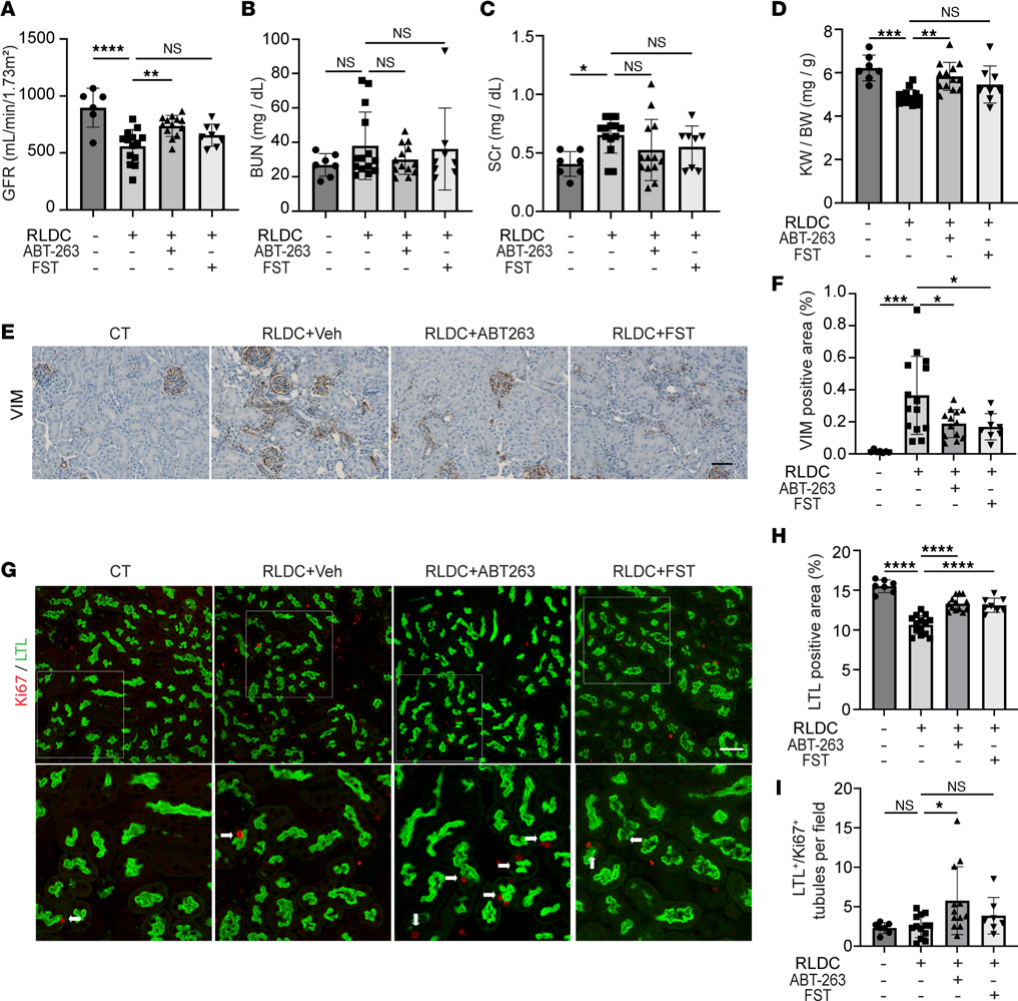
ABT-263 and FST improve renal function and tubular repair in post-RLDC mice. C57BL/6 mice were subjected to RLDC treatment followed by 4-week treatment of ABT-263 (*n* = 13), FST (*n* = 8), or vehicle (*n* = 14). Control (CT) mice (*n* = 7) were injected with saline. Blood and kidney samples were harvested at 8 weeks for renal function and histology analysis. GFR was measured 1 day before tissue harvesting. (**A–D**) GFR, BUN, SCr, and kidney weight to body weight ratio (KW/BW). (**E**) Representative images of VIM staining. Scale bar: 50 μm. (**F**) Quantification of VIM staining. Positive signals from blood vessels and glomeruli were excluded. (**G**) Representative images of costaining of Ki67 (red) and LTL (green). The bottom images are enlarged from the boxed areas in the top images. Original magnification, ×200 (original); ×400 (enlarged). Arrows indicate regenerative tubules positive for LTL and with Ki67-positive nuclei. Scale bar: 50 μm. (**H**) Quantification of LTL-positive staining areas. (**I**) Quantification of Ki67^+^/LTL^+^ regenerative tubules. Quantitative data are presented as mean ± SD. One-way ANOVA with multiple comparisons was used for statistics. *P < 0.05, **P < 0.01, ***P < 0.005, and ****P < 0.001.

Next, we examined the effects of senolytics on renal function and tubular regeneration. RLDC reduced glomerular filtration rate (GFR) from an average of 896 mL/min/1.73m^2^ in control mice to 560 mL/min/1.73m^2^. The decline of GFR in post-RLDC mice was significantly reversed by ABT-263, with the value increased to 737 mL/min/1.73m^2^. FST also improved GFR, although the effect was less obvious (Figure 3A). Post-RLDC mice did not show increased blood urea nitrogen (BUN) at the time point we observed (Figure 3B), but they had a consistent, moderate induction of serum creatinine (SCr) (Figure 3C). Both ABT-263 and FST marginally suppressed the elevation of SCr, although the effects did not reach statistical significance (Figure 3C). In line with tubular atrophy and nephron loss in cisplatin-induced CKD, the kidney/body weight ratio (KW/BW) was decreased in post-RLDC mice as compared with control mice (Figure 3D). ABT-263 largely reversed kidney shrinkage and restored KW/BW. FST was less effective but also partially inhibited the decrease of KW/BW (Figure 3D). These data indicate that persistent senescence contributes to kidney degeneration and the decline of renal function in post-RLDC mice.

We further examined tubular histology. Vimentin (VIM), a tubular dedifferentiation marker, was mainly seen in the glomeruli of control mice, with scattered staining in some interstitial cells. Following RLDC, the expression of VIM was increased, especially in the cells of atrophic tubules, indicating tubular dedifferentiation and degeneration (Figure 3, E and F). Both ABT-263 and FST diminished tubular expression of VIM but had no significant effect on interstitial or glomerular VIM expression (Figure 3, E and F), suggesting that senolytic drugs may promote tubular redifferentiation in post-RLDC mice. Moreover, RLDC also led to a sustained brush border loss in proximal tubules, reducing the areas of lotus tetragonolobus lectin (LTL) staining from 15.51% in control mice to 10.64% in mice after RLDC treatment (Figure 3, G and H). Both ABT-263 and FST blocked the chronic tubular injury in post-RLDC mice, partially but significantly improving the LTL-positive staining areas back to approximately 13% (Figure 3, G and H). Costaining of LTL with Ki67 showed that the LTL and Ki67 double-positive (LTL^+^/Ki67^+^) tubules were rarely seen in vehicle-treated post-RLDC mice, whereas they were significantly increased in ABT-263–treated post-RLDC mice (Figure 3, G and I), further suggesting a recovery of tubular regeneration and adaptive repair after the clearance of chronic senescent tubular cells in post-RLDC mice.

### RLDC induces senescence in cultured mouse proximal tubular cells (BUMPT).

**Figure 4:**
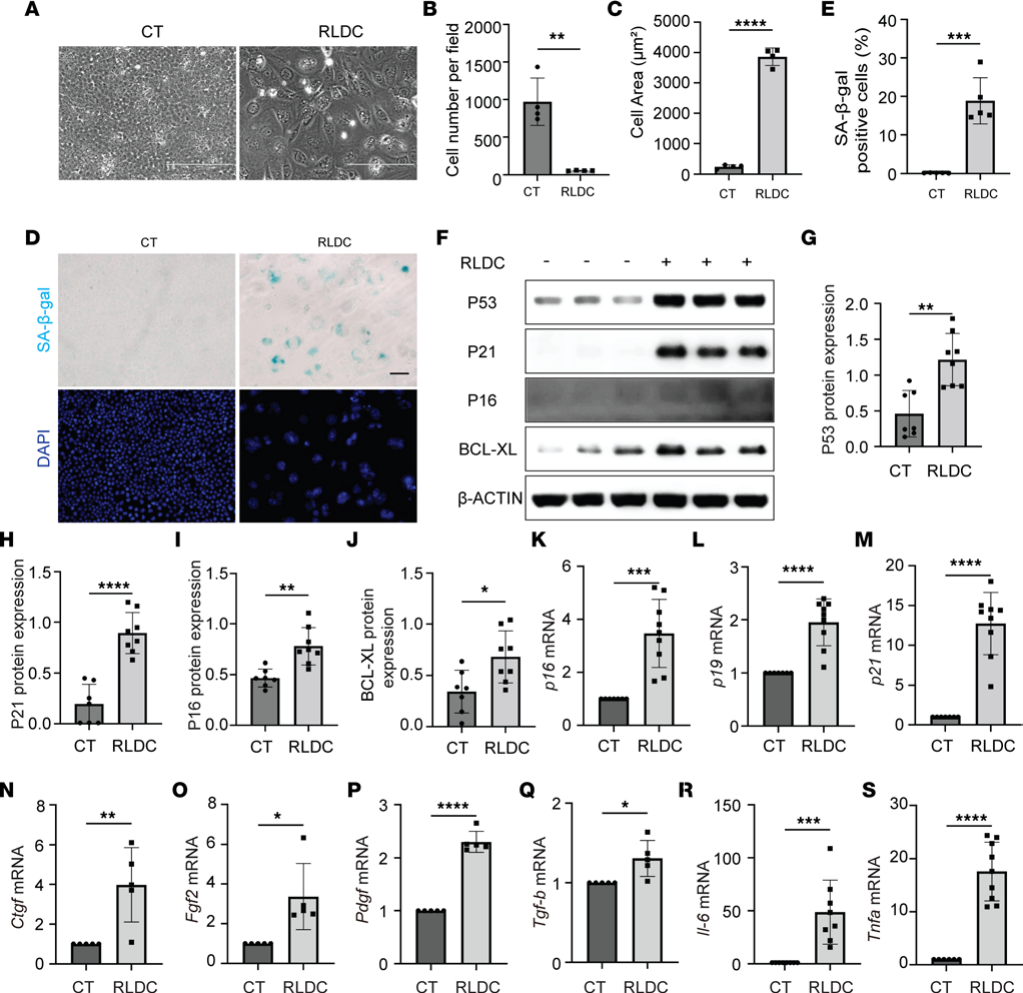
RLDC induces senescence in cultured mouse renal proximal tubular (BUMPT) cells. BUMPT cells were subjected to RLDC treatment or kept in control (CT) medium for 96 hours. Cells were collected for senescence analysis. (**A**) Cell morphology was recorded by phase contrast microscopy. Scale bar: 200 μm. (**B** and **C**) Quantification of the numbers and sizes of BUMPT cells (*n* = 4 experiments). (**D**) Representative images of costaining of SA-β-gal (top panels) and DAPI (bottom panels). Scale bar: 50 μm. (**E**) Quantification of SA-β-gal–positive cells (*n* = 5 experiments). (**F**) Immunoblot analysis for P53, P21, P16, and BCL-XL. (**G–J**) Densitometry of P53, P21, P16, and BCL-XL expression (*n* = 7 experiments). (**K–S**) RT-PCR analysis of *p16*, *p19*, *p21*, *Ctgf*, *Fgf2*, *Pdgf*, *Tgfb*, *Il-6,* and *Tnfa* mRNA expression (*n* = 5 experiments). Quantitative data are presented as mean ± SD. Two-tailed, unpaired *t* test was used for statistics. *P < 0.05, **P < 0.01, ***P < 0.005, and ****P < 0.001.

To understand how tubular senescence induces interstitial fibrosis, we used the in vitro model, in which BUMPT cells were exposed to 4 cycles of 2 μΜ cisplatin treatment, with each cycle containing a 7-hour cisplatin followed by a 17-hour cisplatin-free culture medium incubation (30). Compared with control BUMPT cells that formed cobblestone monolayers typical of epithelium with intact tight junctions, post-RLDC BUMPT acquired a “flattened” appearance mixed with spindle-shape cells (Figure 4A). RLDC increased the size and volume of individual cells but largely inhibited cell proliferation, leading to a remarkable reduction in cell numbers (Figure 4, B and C). SA-β-gal–positive staining was barely seen in control cells, whereas it was significantly increased in post-RLDC cells (Figure 4D, SA-β-gal). Quantitatively, the percentage of senescent BUMPT cells was induced from 0.14% in control group to 18.83% by RLDC (Figure 4E). Moreover, in the majority of the cells with increased SA-β-gal activity, there were enlarged, irregularly shaped nuclei with marked senescence-associated heterochromatin foci (SAHF) formation (Figure 4D, DAPI). Consistent with the typical morphology of cellular senescence, the expressions of several senescence-regulating genes, including *p16, p19, p21*, and *p53*, were all significantly upregulated in post-RLDC BUMPT cells at mRNA and/or protein levels (Figure 4, F–I and K–M). BCL-XL, a well-known antiapoptotic protein, was also induced by RLDC in BUMPT cells, consistent with the apoptosis resistance of senescent cells (Figure 4, F and J). Multiple SASP factors, including *Ctgf*, *Fgf2*, *Pdgfb*, *Tgfb*, *Il-6*, and *Tnfa*, were also increased in BUMPT cells following RLDC (Figure 4, N–S), further indicating the induction of tubular cell senescence in this in vitro model.

### ABT-263 selectively kills senescent BUMPT cells induced by RLDC.

**Figure 5:**
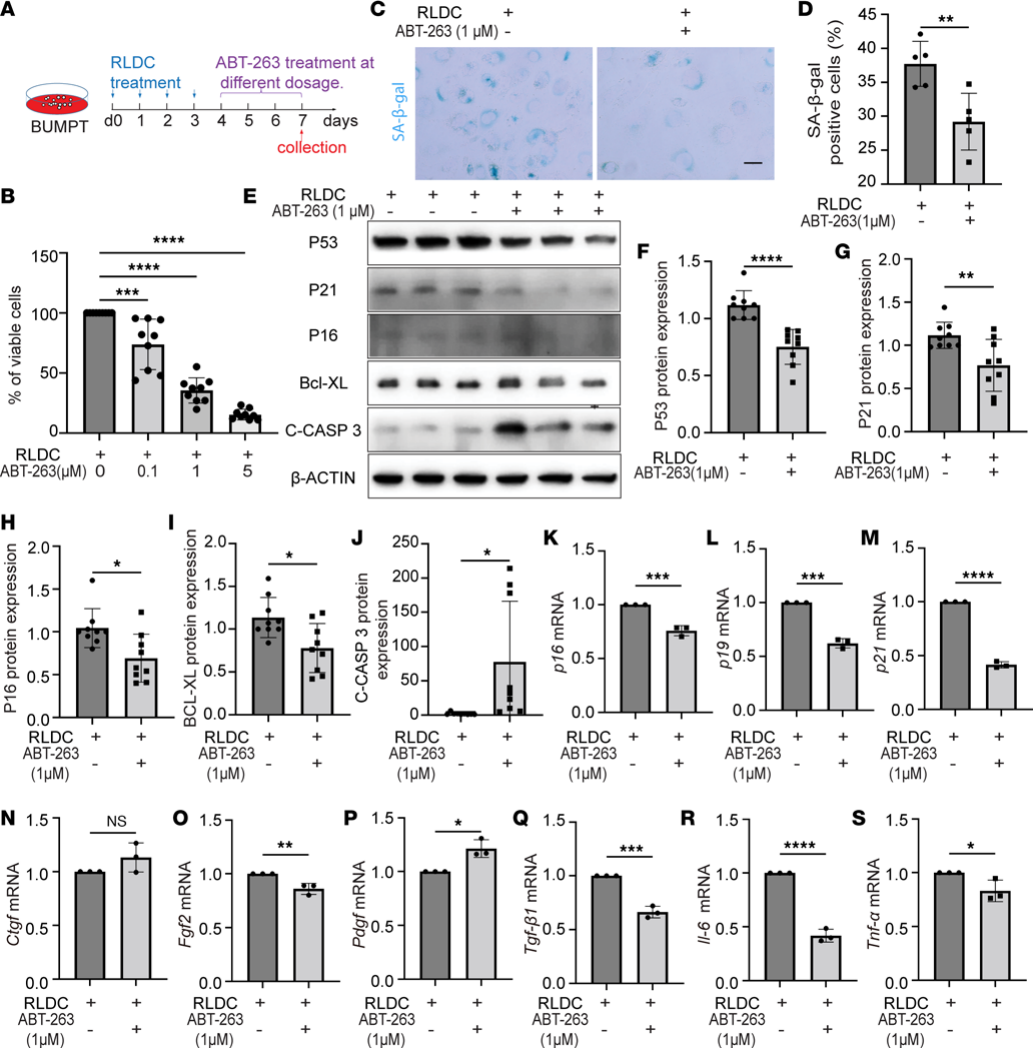
ABT-263 selectively kills senescent BUMPT cells induced by RLDC. (**A**) Schematic diagram of treatment. BUMPT cells were subjected to RLDC treatment followed by 3-day treatment of ABT-263 or vehicle for senescence, immunoblotting, and RT-PCR analysis. (**B**) Quantification of the percentage of viable cells (*n* = 9 experiments). The values of the RLDC + ABT-263 groups are normalized by the RLDC group. (**C**) Representative image of SA-β-gal staining. Scale bar: 50 μm. (**D**) Quantification of SA-β-gal–positive cells (*n* = 5 experiments). (**E**) Immunoblot analysis for P53, P21, P16, BCL-XL, and C-CASP 3. (**F–J**) Densitometry of P53, P21, P16, BCL-XL, and C-CASP 3 expression (*n* = 9 experiments). (**K–S**) RT-PCR analysis of *p16*, *p19*, *p21*, *Ctgf*, *Fgf2*, *Pdgf*, *Tgfb*, *Il-6,* and *Tnfa* mRNA expression. For statistics, 1-way ANOVA with multiple comparisons was used for (**B**); 2-tailed, unpaired t test was used for (**D** and **F–S**). Quantitative data are presented as mean ± SD. *P < 0.05, **P < 0.01, ***P < 0.005, and ****P < 0.001.

With better senolytic effects in post-RLDC mice, ABT-263 was chosen for the in vitro studies. As a BH3 mimic, ABT-263 targets the BCL-2 family members, including BCL-2, BCL-W, and BCL-XL, to induce apoptosis specifically in senescent cells (31). BUMPT cells were subjected to RLDC followed by 3-day treatment with either vehicle or ABT-263 at different concentrations of 0.1, 1, and 5 μM (Figure 5A). The cell-killing effect of ABT-263 was dose dependent (Figure 5B), and we used 1 μM in subsequent experiments. As shown in Figure 5, C and D, ABT-263 significantly decreased the number of senescent cells following RLDC exposure, reducing the percentage of SA-β-gal–positive cells from 38% in the vehicle-treated group to 29%. The increased mRNA and/or protein expressions of senescence-regulating genes, including *p16*, *p19*, *p21*, and *p53*, in post-RLDC BUMPT cells were all suppressed by ABT-263 (Figure 5, E–H and K–M). Notably, these changes were accompanied with caspase-3 activation (shown by C-CASP3) and decreased BCL-XL expression, indicating the induction of apoptosis in senescent BUMPT cells by ABT-263 (Figure 5, E, I, and J). ABT-263 also reduced the mRNA expression of multiple SASP factors in post-RLDC BUMPT cells (Figure 5, N–S), further confirming its inhibitory effects on tubular cell senescence in this in vitro model.

### ABT-263 suppresses RLDC-induced fibrotic phenotype transition and increases the proliferative and clonogenic activity of BUMPT cells.

**Figure 6:**
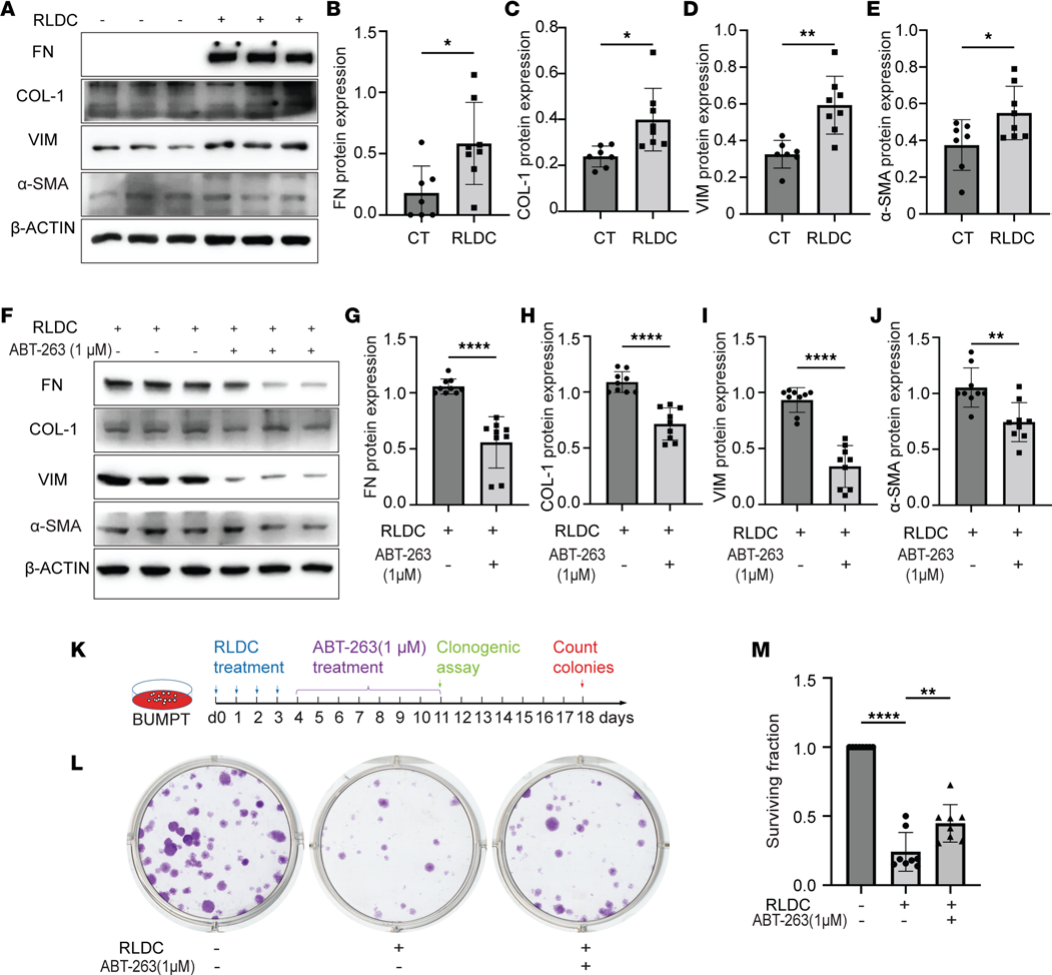
ABT-263 suppresses RLDC-induced fibrotic phenotype and increases clonogenic activity of BUMPT cells. (**A–E**) BUMPT cells were subjected to RLDC treatment or kept in control (CT) medium for 96 hours. Cell lysates were collected for immunoblot analysis of FN, COL1, VIM, and α-SMA (**A**) and densitometry (**B–E**) (*n* = 7 experiments). (**F–J**) BUMPT cells were subjected to RLDC treatment followed by 3-day treatment with or without ABT-263. Cell lysates were collected for immunoblot analysis of FN, COL1, VIM, and α-SMA (**F**) and densitometry (**G–J**) (*n* = 9 experiments). (**K**) The schematic diagram of treatment. BUMPT cells were subjected to RLDC treatment followed by 7-day treatment of ABT-263 or vehicle. Cells were collected and re-seeded for clonogenic assay. (**L**) Representative images showing the colonies originated from initial 200 cells/well for each group. Untreated proliferating BUMPT cells were used as a normal control. (**M**) Quantification of surviving fractions (*n* = 8 experiments). Quantitative data are presented as mean ± SD. For statistics, 2-tailed, unpaired t test was used for (**B–E** and **G–J**); 1-way ANOVA with multiple comparisons was used for (**M**). *P < 0.05, **P < 0.01, ***P < 0.005, and ****P < 0.001.

Along with the induction of tubular cell senescence, we also observed a fibrotic phenotype in post-RLDC BUMPT cells, as indicated by increased expression of several fibrosis markers, including FN, COL1, VIM, and α-SMA (Figure 6, A–E). Again, ABT-263 significantly inhibited the expression of these proteins in post-RLDC BUMPT cells (Figure 6, F–J), supporting our in vivo findings that sustained tubular cell senescence contributes to the profibrotic phenotype transition of tubular cells, leading to maladaptive repair and the progression of cisplatin-induced CKD.

To determine the effect of senolysis on tubular cell proliferation in the in vitro model, we extended the duration of ABT-263 treatment to 7 days after the completion of RLDC (Supplemental Figure 3A) and examined the regrowth of surviving BUMPT cells in situ during ABT-263 incubation. Post-RLDC BUMPT appeared 70%–80% confluent before senolysis treatment (Supplemental Figure 3B, D4), which was significantly reduced to 20%–30% confluent following the first 3 days of ABT-263 exposure (Supplemental Figure 3B, ABT-263 +, D7). A sparse population of surviving BUMPT cells was seen at this time point, with no obvious cell proliferation (Supplemental Figure 3B, ABT-263 +, D7). Of interest, the surviving post-RLDC cells started to proliferate on day 4 of ABT-263 treatment. A few numbers of small but visible cell colonies were formed and scattered throughout culture dishes (Supplemental Figure 3B, ABT-263 +, D8). By the end of the 7-day ABT-263 treatment, the isolated cell colonies expanded and eventually merged into large cell islands to fill up many of the empty areas (Supplemental Figure 3B, ABT-263 +, D11). Notably, the regrowth of surviving post-RLDC cells also exhibited cobblestone monolayers typical of epithelium with intact tight junctions, suggesting a full recovery of tubular structural integrity (Supplemental Figure 3B, ABT-263 +, D11). By contrast, the proliferation of surviving post-RLDC cells in vehicle-treated group was largely delayed and slowed down due to the presence of senescent cells. The formation of cell colonies was not detected until the end of 7-day vehicle treatment, smaller in size and fewer in number as compared with ABT-263–treated cells (Supplemental Figure 3B, ABT-263 -). We further conducted a clonogenic assay of surviving BUMPT cells upon the completion of 7-day senolysis treatment (Figure 6K). Compared with normal BUMPT cells that formed numerous big clusters of colonies, the proliferative capacity of post-RLDC BUMPT cells was markedly suppressed (Figure 6L). Notably, ABT-263 rescued post-RLDC cells from growth inhibition, increasing the formation of cell colonies both in size and in number (Figure 6L). Quantitatively, post-RLDC cells only had 25% of the proliferative capacity of normal BUMPT cells, which was restored to 48% by ABT-263 (Figure 6M). These results are consistent with our in vivo findings, suggesting that persistent senescence inhibits tubular regeneration and ABT-263 partially restores it.

### Knockdown of p16 suppresses RLDC-induced tubular cell senescence and fibrotic phenotype transformation.

**Figure 7:**
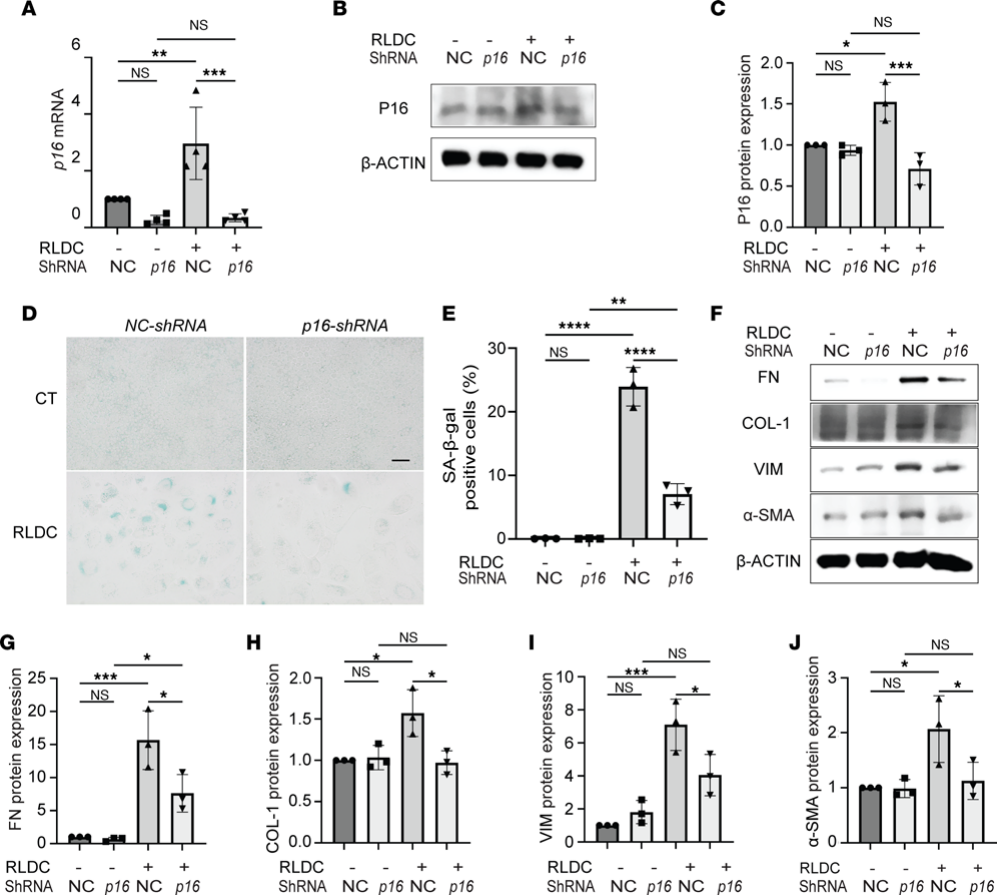
Knockdown of p16 suppresses RLDC-induced tubular cell senescence and fibrotic phenotype transformation. BUMPT cells were transfected with *p16*-shRNA or negative control (NC) shRNA and then selected with puromycin for 2 weeks to establish stable cells for the subsequent RLDC treatment. (**A**) RT-PCR analysis of *p16* mRNA expression (*n* = 4 experiments). (**B** and **C**) Immunoblot and densitometry analysis for P16 protein (*n* = 3 experiments). (**D**) Representative images of SA-β-gal staining. Scale bar: 50 μm. (**E**) Quantification of SA-β-gal–positive cells (*n* = 3 experiments). (**F**) Immunoblot analysis for FN, COL1, VIM, and α-SMA. (**G–J**) Densitometry of FN, COL1, VIM, and α-SMA expression (*n* = 3 experiments). Quantitative data are presented as mean ± SD. Two-way ANOVA with multiple comparisons was used for statistics. *P < 0.05, **P < 0.01, ***P < 0.005, and ****P < 0.001.

To further verify the role of tubular cell senescence in cisplatin-induced CKD, we transfected BUMPT cells with *p16* shRNA and examined the effects of genetic targeting senescence on RLDC-induced tubular pathologies. P16 was induced by RLDC at mRNA and protein levels in negative control (NC) shRNA–transfected cells, both of which were significantly inhibited by *p16* shRNA (Figure 7, A–C). Knockdown of *p16* remarkably suppressed tubular cell expression of SA-β-gal following RLDC treatment, reducing the percentage of SA-β-gal–positive cells from approximately 25% in NC shRNA group to approximately 7% (Figure 7, D and E). The induction of multiple senescence-regulating genes and SASP factors during RLDC treatment was also attenuated in *p16* shRNA cells as compared with NC shRNA cells (Supplemental Figure 4). Moreover, RLDC induced fibrotic changes in NC shRNA–transfected BUMPT cells, as indicated by the increased expression of multiple fibrosis markers including FN, COL1, VIM, and α-SMA (Figure 7, F–J, NC shRNA). Consistent with the senolytic drug ABT-263, genetic inhibition of tubular cell senescence by *p16* shRNA significantly inhibited tubular fibrotic phenotype transition (Figure 7, F–J, p*16* shRNA), providing further in vitro evidence to support the role of sustained tubular cell senescence in promoting tubular pathologies for maladaptive repair and the progression of cisplatin-induced CKD.

### Both pharmacologic and genetic inhibition of senescence alleviates the paracrine effect of senescent tubular cells on fibroblast proliferation.

**Figure 8:**
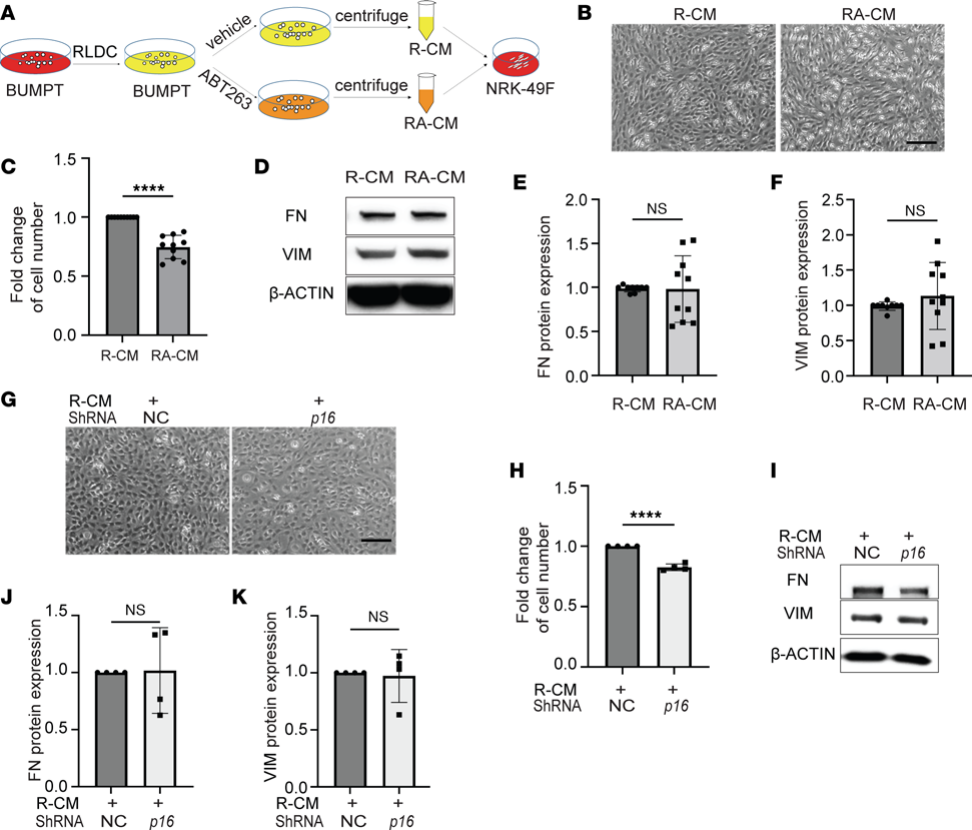
Both pharmacologic and genetic inhibition of senescence alleviates the paracrine effect of senescent tubular cells on fibroblast proliferation. (**A**) Schematic diagram of treatment. Conditioned medium was collected from either post-RLDC BUMPT cells (R-CM) or post-RLDC BUMPT cells treated with ABT-263 (RA-CM). NRK-49F fibroblasts were incubated with serum-free medium containing R-CM or RA-CM for 48 hours for morphological and immunoblot analysis (*n* = 10 experiments). (**B**) Cell morphology of NRK-49F. Scale bar: 400 μm. (**C**) Quantification of the fold changes of NRK-49F fibroblast cell numbers. (**D**) Immunoblot analysis for FN and VIM in NRK-49F fibroblasts. (**E** and **F**) Densitometry of FN and VIM expression. (**G–K**) Conditioned medium was collected from post-RLDC BUMPT cells stably transfected with *p16*-shRNA (*p16–*R-CM) or negative control shRNA (NC–R-CM). NRK-49F fibroblasts were incubated with serum-free medium containing NC–R-CM or *p16–*R-CM for 48 hours for morphological and immunoblot analysis (*n* = 4 experiments). (**G**) Cell morphology of NRK-49F. Scale bar: 400 μm. (**H**) Quantification of the fold changes of NRK-49F fibroblast cell numbers. (**I**) Immunoblot analysis for FN and VIM in NRK-49F fibroblasts. (**J** and **K**) Densitometry of FN and VIM expression. Quantitative data are presented as mean ± SD. For statistics, 2-tailed, unpaired t test was used. *P < 0.05, **P < 0.01, ***P < 0.005, and ****P < 0.001.

In maladaptive kidney repair, injured renal tubules can drive disease progression via releasing profibrotic and proinflammatory cytokines to activate themselves by autocrine or neighboring cells by paracrine mechanisms (5–10). Our recent work demonstrates that autophagic tubular cells produces FGF2 after kidney injury to activate fibroblasts for maladaptive repair and kidney fibrosis (29). To determine whether RLDC-induced senescent BUMPT cells can activate renal fibroblasts in a paracrine manner, we collected conditioned medium (CM) from both control (C-CM) and post-RLDC (R-CM) BUMPT cells to treat renal NRK-49F fibroblasts (Supplemental Figure 5A). The secretion of profibrotic cytokines such as CTGF and FGF2 in R-CM was confirmed by immunoblot analysis (Supplemental Figure 5B). Compared with C-CM, R-CM significantly promoted fibroblast proliferation and activation, as indicated by increased cell number (Supplemental Figure 5, C and D) and elevated expression of FN and VIM in NRK-49F fibroblasts (Supplemental Figure 5, E–G), suggesting the paracrine effect of senescent tubular cells on renal fibroblasts in cisplatin-induced CKD. Notably, addition of FGF2 neutralizing antibody appeared to partially block the communication between senescent tubular cells and renal fibroblasts. R-CM–induced fibroblast proliferation was alleviated by the neutralizing antibody (Supplemental Figure 5, H and I), whereas fibroblast activation, particularly the increased production of FN, was not affected (Supplemental Figure 5, J–L). Along with our recent work (29), these results suggest that FGF2 may be one of the many SASP factors produced by chronically senescent renal tubular cells to mediate fibroblast proliferation and activation during maladaptive repair in different post-AKI models, including cisplatin nephrotoxicity. To further determine the role of senescence, we collected CM from post-RLDC BUMPT cells (R-CM) and post-RLDC BUMPT cells treated with ABT-263 (RA-CM) to incubate NRK-49F fibroblasts (Figure 8A). Fibroblast proliferation induced by RA-CM was significantly less than that induced by R-CM (Figure 8, B and C), whereas fibroblast activation seemed indifferent (Figure 8, D–F). These data suggest that ABT-263 may partially alleviate the paracrine effect of senescent tubular cells on fibroblasts to block the progression of cisplatin-induced CKD. Consistent with the pharmacological results, CM collected from post-RLDC BUMPT cells transfected with *p16* shRNA (*p16–*R-CM) was also less effective in stimulating fibroblast proliferation compared with CM from NC shRNA–transfected cells (NC–R-CM) (Figure 8, G–K), further supporting the role of tubular cell senescence in paracrine activation of renal fibroblasts to promote maladaptive repair during cisplatin-induced CKD.

## Discussion

**Figure 9:**
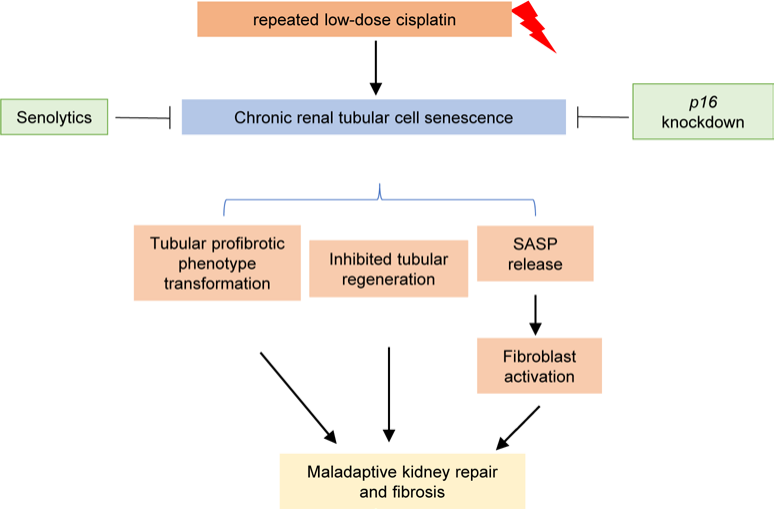
Graphic summary highlighting the pathological role of chronic tubular cell senescence in cisplatin-induced CKD and the therapeutic potential of senolytics.

Nephrotoxicity is a main limiting side effect for the use of cisplatin in cancer therapy. Although a lot is known about cisplatin-induced AKI, the mechanism of maladaptive kidney repair and CKD after cisplatin chemotherapy is largely unclear. In this study, using both in vivo and in vitro models of RLDC treatment, we have demonstrated a pathological role of renal tubular cell senescence in cisplatin-induced CKD progression. In our study, sustained cellular senescence was induced in renal tubules of post-RLDC mice and in RLDC-treated mouse proximal tubular cells (BUMPT), leading to tubular degeneration and transition to a fibrotic phenotype with persistent production of various profibrotic factors and increased expression of fibrotic markers. These chronic tubular pathologies disturbed tubular regeneration and also activated renal fibroblasts to promote interstitial fibrosis. Notably, clearance of senescent tubular cells by senolytics attenuated kidney fibrosis, rejuvenated tubular proliferation, and partially restored renal function, further suggesting a therapeutic potential of targeting tubular senescence to protect the kidney from chronic disease during cisplatin chemotherapy (Figure 9).

Cisplatin-induced renal tubular senescence has been reported recently. In cultured rat renal tubular epithelial cells (NRK-52E), cisplatin treatment at 20 μM for 6 hours leads to a time-dependent induction of senescence within 72 hours (32). In mice, a single dose of cisplatin induces SA-β-gal in renal tubules 28 days after the injection (33). Mice treated with 3 doses of cisplatin at 10 mg/kg also exhibit hallmark changes of senescence in renal tubules (32, 34). Compared with previous studies, the in vivo and in vitro RLDC models used in our current study consisted of 4 doses of cisplatin at relatively lower concentrations (8 mg/kg in mice and 2 μM in BUMPT cells). Under these conditions, we also revealed that RLDC increased senescence in cultured BUMPT cells and in mouse renal tubules. Of note, in studies using different RLDC regimen to investigate the long-term effects of cisplatin on the kidney, there are noticeable differences in terms of the severity of tubular injury, the extent and duration of renal function loss, and the development of interstitial fibrosis (30). Nonetheless, the induction of tubular senescence appears to be a common event and key pathological feature of cisplatin-induced CKD in different experimental settings, which makes senescence a potential therapeutic target for renoprotection during cisplatin treatment. Indeed, we showed that by killing senescent tubular cells, both ABT-263 and FST improved kidney repair and suppressed cisplatin-induced CKD. Consistent with our findings, Li et al. recently demonstrated that senolytic combination therapy with Dasatinib and Quercetin also ameliorates kidney fibrosis and disease progression in different post-AKI models including cisplatin nephrotoxicity (34).

Depending on the timing of induction and the duration, tubular cell senescence may play different roles in AKI and kidney repair after AKI (18–20). For example, in a mouse model of renal artery stenosis-induced chronic renal ischemia, inhibition of senescence within the first week after ischemia hinders functional recovery, whereas senolytics treatment started from 2 weeks after ischemia reduces tubular damage and improves kidney function (24). Therefore, delineating the time course of kidney injury and dissecting the different roles of tubular senescence in injury and repair phases are important for identifying a therapeutic time window to target senescence. Compared with the renal ischemia/reperfusion model, the phases of injury and repair in the RLDC model are less defined. To characterize this model, our recent studies have examined the morphological and functional changes at different time points in post-RLDC mice. The results suggest that the worst kidney injury occurs within the first week after the completion of RLDC and recovery begins thereafter as indicated by regaining renal function and body weight (27, 30). Given these data, here we monitored senescence in kidneys at 1 week and 5 weeks after the completion of RLDC treatment in mice, timing the changes of senescence at the different stages of kidney repair and disease progression. We found that senescence persisted in post-RLDC renal tubules for weeks, accompanied with the development of CKD. We also chose this time window for senolytic treatment to specifically target senescence during the repair phase without comprising its potential protective effects during the injury phase.

The mechanisms underlying prolonged tubular cell senescence in the context of AKI to CKD transition including cisplatin nephrotoxicity are being investigated. In this regard, the inhibition of cisplatin-induced tubular senescence by N-acetylcysteine is dependent on SIRT1/P53, indicating a potential role of this signaling pathway in promoting senescence (32). A p53-dependent pathway is also shown to be crucial for cellular senescence induction and long-term outcome after renal ischemia/reperfusion (35). Notably, using renal tubule–specific autophagy gene knockout models, we and others have demonstrated a persistent induction of autophagy in renal tubules in post-ischemic AKI mice, which may promote tubular cell senescence for maladaptive repair (28, 29). In a post-AKI model induced by folic acid, tubular cell senescence is mediated by epithelial Toll-like and IL-1 receptors of the innate immune system (33). Tubular cell–specific inhibition of innate immune signaling in mice by knockout of myeloid differentiation 88 (Myd88) prevents the accumulation of senescent tubular cells, suggesting a cell-autonomous role for epithelial innate immunity in controlling cell senescence after kidney injury (33). Moreover, in aging kidneys after ischemic AKI, NOTCH1 is activated and sustained in renal tubular cells, leading to a prosenescent phenotype and maladaptive repair (36). These findings are of particular interest, as recent studies suggest that NOTCH1 is an essential driver of oncogene-induced secondary senescence for the accumulation of senescent cells (37, 38). With the emergence of novel insights, further in-depth investigations are much needed.

In our present study, clearance of senescent tubular cells not only attenuated interstitial fibrosis in post-RLDC mice, but, more importantly, it improved tubular repair, as indicated by the restoration of both tubular regeneration and renal function. Consistently, after rescue by senolytic drug ABT-263, post-RLDC BUMPT cells also acquired a better proliferative and clonogenic activity. It is unclear how tubular cell regeneration is restored following the elimination of senescent tubular cells. Interestingly, we found that after 3-day treatment of ABT-263 (1 μM) in post-RLDC BUMPT cells, over 50% of senescent tubular cells died by apoptosis, leaving large areas of empty space in culture dishes. On day 4 after RLDC, the surviving cells started to proliferate irrespective of the continuous presence of ABT-263 for up to 7 days after RLDC. Given these observations, we speculate that elimination of senescent tubular cells could provide space for surviving tubular cells to proliferate as contact inhibition is abolished. Recently, Ritschka et al. uncovered a timely function of the SASP in promoting proregenerative response (39). They showed that primary mouse keratinocytes transiently exposed to the SASP exhibited increased expression of stem cell markers and regenerative capacity, whereas prolonged exposure to the SASP caused a paracrine senescence to neighboring nonsenescent cells to counter regeneration (39). These results suggest the importance of a microenvironment with restricted SASP to tissue regeneration. In line with this, Bird et al. reported that inhibition of paracrine hepatocellular senescence after acute liver injury restored liver regeneration (40). Whether these mechanisms contribute to the restoration of tubular regeneration following the clearance of senescent tubular cells in the settings of AKI to CKD transition awaits further investigation.

## Methods

### Antibodies and reagents

#### Primary antibodies.

Anti–BCL-XL (BD-610209) was from BD Biosciences; anti-CTGF (NBP2-16026) and anti-COL1 (NBP1-30054, NB-600408) were from Novus Biologicals; anti-FN (ab2413), anti–α-SMA (ab5694), anti-P16 (ab189034, ab211542), and anti-γH2A.X (ab26350) were from Abcam; anti-VIM (3932), anti–cleaved caspase-3 (9664), anti-P53 (2524, 32532), anti-Ki67 (9129), anti–β-actin (3700), and anti-GAPDH (2118) were from Cell Signaling Technology; anti-P21 (AHZ0422) was from Invitrogen; and anti-FGF2 (05-118) and FGF2 neutralizing antibody (05-118) were from MilliporeSigma. Secondary antibodies for immunoblot analysis were from Jackson ImmunoResearch Laboratories. Cisplatin (P4394) and crystal violet (C0775) were from MilliporeSigma. ABT-263 (201970) was from MedKoo Biosciences. FST (528-48-3) was from Selleck Chemicals.

### Mouse model of RLDC treatment and test of senolytic drugs

C57BL/6 mice were originally purchased from The Jackson Laboratory and housed in a pathogen-free animal facility of Charlie Norwood VA Medical Center under a 12/12-hour light/dark pattern with free access to food and water.

RLDC treatment in mice to induce CKD was described in our recent work (27). Briefly, 12-week-old male C57BL/6 mice were subjected to weekly injection of cisplatin at 8 mg/kg for 4 consecutive weeks. Kidney tissues and blood samples were collected at 4 or 8 weeks after the first injection for further analysis.

For senolytic treatment, 1 week after the completion of RLDC, mice were given intraperitoneal injection of either vehicle (10% DMSO + 40% Polyethylene glycol 300 + 5% Tween80 in saline) or the senolytic drugs (ABT-263 at 37.5 mg/kg; FST at 100 mg/kg) for 4 cycles. Each cycle contained 5 consecutive daily injection and a subsequent 2-day interval. The dosages of ABT-263 and FST were described in previous studies (41, 42) with some modifications according to our pilot tests. Mice were monitored daily for body weight and overall health. The numbers of animals used in each group: control or control + vehicle (*n* = 7), control + ABT-263 (*n =* 5), control + FST (*n* = 5), RLDC + vehicle (*n* = 14), RLDC + ABT-263 (*n =* 13), RLDC + FST (*n* = 8). Upon the completion of senolytic treatment, kidney tissues and blood samples were collected for further analysis.

### Cell lines

The immortalized mouse renal proximal tubule cell line (BUMPT) was originally obtained from Wilfred Lieberthal (Boston University School of Medicine, Boston, Massachusetts, USA). Rat renal fibroblast cell line (NRK-49F) was purchased from the ATCC. Both cells were maintained in DMEM medium containing 10% FBS.

To generate *p16*-knockdown mouse proximal tubular cells, MISSION Lentiviral shRNA targeting mouse *p16* (MilliporeSigma, TRCN0000231226 in pLKO-puro vector) was transduced to BUMPT cells following the manufacturer’s protocol. Negative control BUMPT cells were transduced with TRC1 pLKO–puro empty vector. The cells were then selected with 2.5 μg/mL puromycin for 2 weeks to have stable *p16* knockdown cells. The efficiency of gene silencing was confirmed by real-time PCR assay of *p16* mRNA and immunoblot analysis of P16 protein.

### Cell model of RLDC treatment and test of senolytic drugs

BUMPT cells were seeded in 35 mm dishes at a density of 0.2 × 10^6^ cells/dish to reach 20%–30% confluence by the next day. The cells were then exposed to 4 cycles of cisplatin at 2 μΜ with each cycle containing a 7-hour cisplatin treatment followed by a 17-hour cisplatin-free culture medium incubation. After treatment, 5 random 200× fields from each dish were captured by Evos microscope with scaling. The cell number and the size of individual cells in each field were analyzed using ImageJ (NIH) as described previously (43). For ABT-263 treatment, post-RLDC BUMPT cells were further exposed to 1 μM ABT-263 for 3 days, with daily refreshment of medium and the drug.

### Collection of tubular cell–conditioned medium for fibroblast treatment

After treatment, BUMPT cells were washed with PBS buffer to remove cisplatin or ABT-263 and maintained in FBS-free DMEM medium for 24 hours. The media were collected and centrifuged at 1,000*g* for 5 minutes to remove cell debris. The supernatants were collected and concentrated using Amicon Ultra-4 centrifugal filter unit with a 3 kDa molecular weight cutoff (UFC8003, MilliporeSigma) according to the manufacturer’s instructions. The tubular cell–conditioned medium (CM) was stored at −80°C until use.

Treatment of NRK-49F fibroblasts with tubular cell–CM was described in our recent work (29). In brief, NRK-49F fibroblasts were seeded in 12-well plates at a density of 0.15 × 10^6^ cells/well. The cells reached approximately 80% confluence by the next day and starved overnight in FBS-free medium. Subconfluent fibroblasts were then incubated for 48 hours with different tubular cell–CM normalized by corresponding BUMPT cell numbers. Cells were monitored morphologically and trypsinized for counting by Bio-Rad TC20 automated cell counter. In experiments testing the effect of FGF2 neutralizing antibody, the antibody or control mouse IgG was preincubated with tubular cell–CM for approximately 1.5 hours at room temperature before adding to NRK-49F fibroblasts. FGF2 neutralizing antibody was tested at different concentrations of 5, 10, and 20 μg/mL.

### SA-β-gal staining

SA-β-gal staining was done with a Senescence Detection Kit (Abcam, ab65351) according to the manufacturer’s instructions. BUMPT cells were cultured on coverslips in 35 mm dishes. After treatment, the cells were fixed with the fixative solution and then incubated with staining solution mix overnight at 37°C. The coverslips were mounted with mounting buffer containing DAPI. For quantification, 5 random 200× fields from each group were selected. Positive cells and nuclei were counted using Adobe Photoshop software and the percentage of SA-β-gal–positive cells was calculated. SA-β-gal staining of kidney tissues was performed on fresh cryosections. Ten random 200× fields were selected for each section for quantification using Image Pro Plus 6.0 software according to the manufacturer’s instructions.

### Clonogenic assay

The proliferation of post-RLDC BUMPT cells was assessed by the clonogenic assay (44). Briefly, post-RLDC BUMPT cells were treated with vehicle or 1 μM ABT-263 for 7 days with daily refreshment of medium and the drug. After treatment, the cells were digested, counted, diluted, and reseeded in 6-well plates at a series of concentration including 100, 200, 400, 800, 1,600, and 3,200 cells per well. Untreated proliferating BUMPT cells were used as normal control. The reseeded cells were further cultured in DMEM medium containing 10% FBS for another 7 days until enough typical cell colonies were observed. The culture medium was refreshed every 2–3 days. Then the colonies were fixed, stained with crystal violet, and counted under the microscope. The clonogenic capacity was quantified by calculating the plating efficiency (PE) and surviving fraction (SF) (44). PE is the ratio of the number of colonies to the number of cells initially seeded, i.e., no. of colonies formed/no. of cells seeded. The number of colonies that arise from cells after treatment, expressed in terms of PE, is called the SF, which can be found from the following formula: SF = no. of colonies formed after treatment/(no. of cells seeded × PE). Higher SF represents higher clonogenic capacity.

### Renal function and transcutaneous measurement of GFR

BUN and SCr were measured using commercial kits (Stanbio Laboratory, 0580 and 0420) according to the manufacturer’s instructions. GFR was measured in mice by monitoring the clearance of FITC-labeled sinistrin using a transcutaneous detector (MediBeacon) as previously described (27). Briefly, mice were anesthetized by isoflurane inhalation, shaved, and depilated to expose the skin of flanks. The transdermal GFR monitors were adhered to the skin using a double-sided adhesive patch. Devices were secured on the mouse by medical tape. FITC-sinistrin (15 mg/mL dissolved in 0.9% sterile saline) was injected at 75 mg/kg via the retro-orbital vein. Mice were placed back in their cages, and GFR was monitored for 1–2 hours. The data were analyzed using the elimination kinetics curve of FITC-sinistrin.

### Histological staining

Kidney tissues were fixed with 4% paraformaldehyde, embedded in paraffin, and sectioned at 4 μm.

#### Sirius Red/Fast Green staining.

Renal fibrosis was examined by Sirius Red/Fast Green staining (Chondrex) according to the manufacturer’s instructions. For quantification, 10 random 100× fields selected from each tissue section and the percentage of positive staining areas was evaluated using ImageJ.

#### Immunohistochemistry.

After rehydration and antigen retrieval, the sections were blocked with 2.5% horse serum before incubation with the following primary antibodies: anti-FN (1:400), anti-COL1 (1:200), anti–α-SMA (1:400), anti-VIM (1:400), and anti–C-CASP3 (1:300). After the incubation with HRP-conjugated secondary antibodies (Vector Laboratories), tissue sections were developed with a DAB Peroxidase Substrate (Vector Laboratories), counterstained with hematoxylin, and mounted. For quantification, 20 random 200× fields were randomly selected from each section and the percentage of positive staining areas or cells was evaluated using Image Pro Plus 6.0 software.

#### Immunofluorescence.

After rehydration and antigen retrieval, the sections were incubated in blocking buffer (2% BSA, 0.2% milk, 0.8% Triton X-100, 2% normal goat serum in PBS). Tissue sections were exposed to anti-γH2A.X (1:200), anti-Ki67 (1:400), anti-P16 (1:150), and anti-KIM-1 (1:400) overnight at 4°C, followed by incubation with Cy3- or FITC-conjugated secondary antibodies. For Ki67 and LTL costaining, the slides were further incubated with LTL for 2 hours at room temperature. The nuclei were counterstained with DAPI. For quantification of γH2A.X/Ki67 costaining, 10 random 630× fields were captured by confocal microscope (Zeiss 780 upright confocal) from each tissue section, and the numbers of γH2A.X-positive (≥4 foci) but Ki67-negative (γH2A.X^+^/Ki67^-^) cells were counted. For quantification of Ki67/LTL costaining, 20 random 200× fields were selected from each tissue section, and the percentage of positive LTL staining areas was evaluated using Image Pro Plus 6.0 software. The numbers of Ki67 and LTL double-positive (Ki67^+^/LTL^+^) tubules were also counted.

### RNA extraction and real-time PCR

Kidney tissue samples were dissected, snap-frozen in liquid N2, and kept at -80°C until use. Total tissue RNA was extracted using a mirVana miRNA Isolation Kit (Thermo Fisher Scientific). BUMPT cell RNA was extracted using a GeneJET RNA Purification Kit (Thermo Fisher Scientific). To measure *Tnfa* mRNA, 2 μg RNA was reversely transcribed using a High-Capacity cDNA Reverse Transcription Kit (Thermo Fisher Scientific), and real-time PCR (RT-PCR) was performed using TaqMan Universal PCR Master Mix (Thermo Fisher Scientific). *Gapdh* was used as an internal control. For the other mRNAs, 1 μg RNA was reversely transcribed using a cDNA Transcription Kit (Bio-Rad), and RT-PCR was performed using SYBR Green PCR Master Mix (Bio-Rad). β*-Actin* was used for normalization. The quantification was done using ΔCt values as recently described (45, 46). Predesigned RT-PCR probes were used for mouse *Tnfa* and mouse *Gapdh*. The sequences of the other primers were shown in Table S1. All primers were synthesized at Integrated DNA Technologies Inc.

### Immunoblot analysis

BUMPT cells and kidney tissues were lysed in 2% SDS buffer (62.5 mM Tris-HCl [pH 6.8], 2% SDS, 10% glycerol) containing protease inhibitor cocktail (MilliporeSigma, P8340) and Benzonase nuclease (MilliporeSigma, 70746). Protein concentration was determined by Pierce BCA protein assay kit (Thermo Fisher Scientific, 23225). Equal proteins were loaded in each lane of for SDS-PAGE electrophoresis and immunoblot analysis using standard methods.

### Statistics

Data are representatives of at least 3 experiments and are expressed as mean ± SD. Statistical analysis was performed using GraphPad Prism software. Significant differences were tested by unpaired, 2-tailed *t* test between 2 groups and by 1-way or 2-way ANOVA with multiple comparisons in multiple groups. A *P* value of less than 0.05 was considered significantly different.

### Study approval

All animal experiments were performed under Institutional Animal Care and Use Committee–approved protocols of Charlie Norwood VA Medical Center.

## Author contributions

SL, ML, HD, DZ, and ZD contributed to the conceptualization, design, and outline of this study; SL performed experiments; SL and ZD interpreted results of experiments; SL and ML prepared the original draft with figures. SL, ML, ZM, XH, LW, HD, DZ, and ZD contributed to the revision and editing. All authors have read the journal’s authorship agreement, reviewed, and approved the manuscript.

## Supplementary Material

Supplemental data

## Version 1 03/14/2023

In-Press Preview

## Version 2 04/24/2023

Electronic publication
