# Supplementary material for: Tubular cell senescence promotes maladaptive kidney repair and chronic kidney disease after cisplatin nephrotoxicity
Source: JCI Insight. 2023 Apr 24;8(8):e166643. doi: 10.1172/jci.insight.166643 (PMC10243740; doi:10.1172/jci.insight.166643)
Supplement: Supplemental data [file jciinsight-8-166643-s166.pdf]

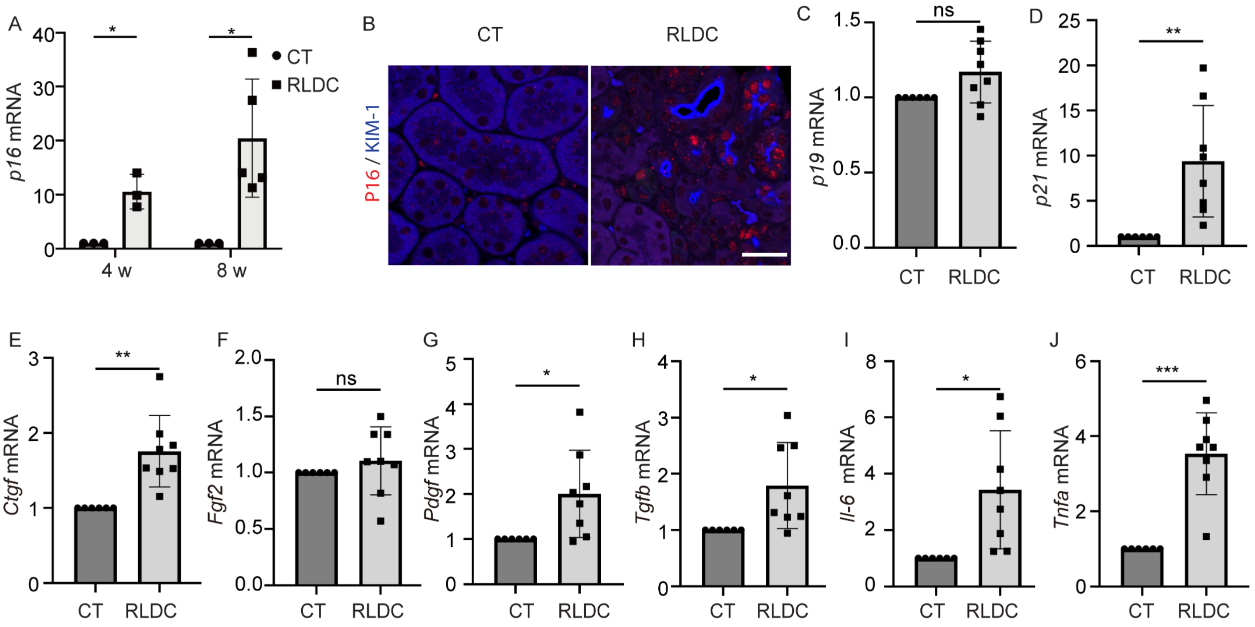


**Supplemental Figure 1. Multiple senescence-regulating genes and SASP factors are induced in post-RLDC kidneys.**

C57BL/6 mice were subjected to RLDC or saline control (CT) treatment. (**A**) Kidneys were collected at both 4 weeks and 8 weeks for RT-PCR analysis of p16 mRNA expression (4 w: CT: n=3, RLDC: n=3; 8 w: CT: n=3, RLDC: n=5). (**B**) Representative images of co-staining of P16 (red) and KIM-1 (blue). Scale bar: 50 μm. (**C-J**) RT-PCR analysis of *p19*, *p21*, *Ctgf*, *Fgf2*, *Pdgfb*, *Tgfb*, *Il-6* and *Tnfa* mRNA expression at 8 weeks (CT: n=6, RLDC: n=8). Quantitative data are presented as mean ± SD. For statistics, two-way ANOVA with multiple comparisons was used for (**A**); 2-tailed, unpaired t test was used for (**C-J**). *P < 0.05, **P < 0.01, ***P < 0.005, and ****P < 0.001.

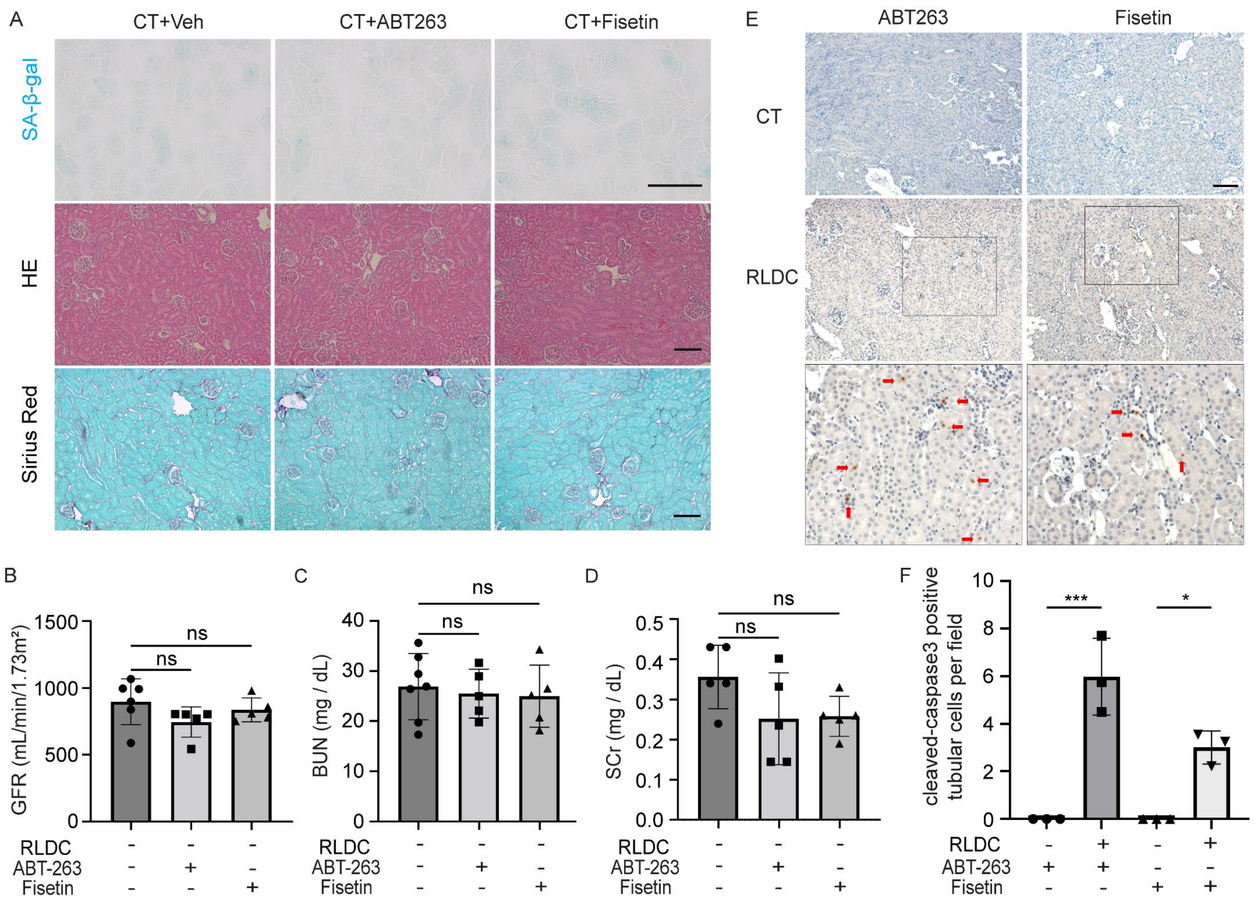


**Supplemental Figure 2. Both ABT-263 and Fisetin induces senescent tubular cell apoptosis in post-RLDC mice whereas not toxic to control mice.**

(**A-D**) Saline control (CT) mice were subjected to 4-week treatment of vehicle (n=5 to 7), ABT-263 (n=5) or Fisetin (n=5). Blood samples and kidneys were harvested for renal function and histology analysis. Glomerular filtration rate (GFR) was measured one day before tissue harvesting. (**A**) Representative images of SA-β-gal staining (top panels), hematoxylin-eosin (HE) staining (middle panels) and Sirius red/Fat Green staining (bottom panels). Scale bar: 50 µm. (**B-D**) Quantification of GFR, blood urea nitrogen (BUN) and serum creatinine (SCr). (**E**) Both saline control (CT) and post-RLDC mice were subjected to 4-week treatment of ABT-263 or Fisetin. Kidneys were collected for immunohistochemical staining of cleaved caspase 3. The bottom images are enlarged from the boxed areas in RLDC groups. Arrows indicate cleaved caspase 3 positive tubular cells. Scale bar: 50 µm. (**F**) Quantification of cleaved caspase 3 positive tubular cells per 400× field (n=3 in each group). Quantitative data are presented as mean ± SD. One-way ANOVA with multiple comparisons was used for statistics. *P < 0.05, **P < 0.01, ***P < 0.005, and ****P < 0.001.

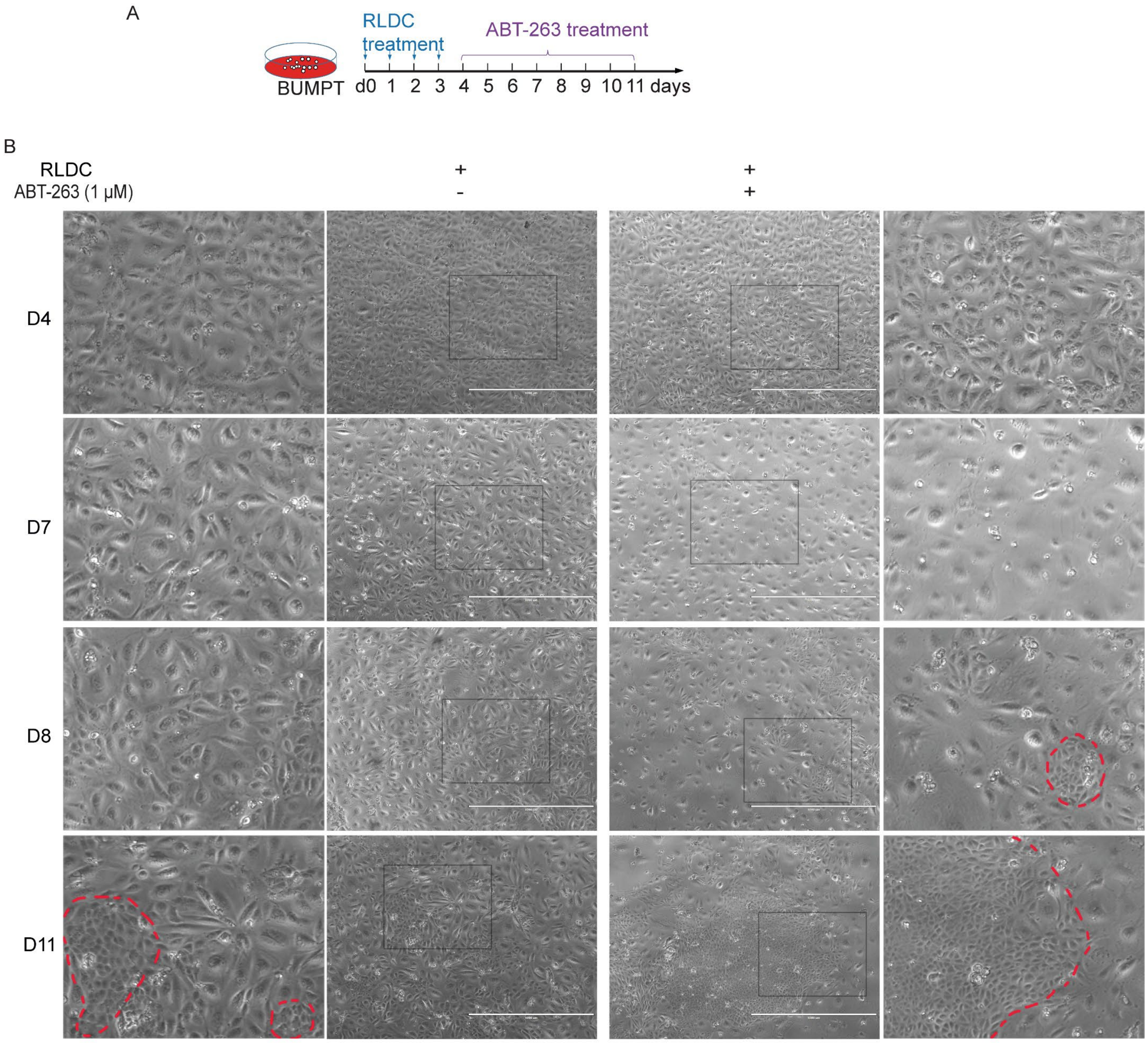


**Supplemental Figure 3 ABT-263 promotes the proliferation of surviving BUMPT cells after RLDC treatment.**

(**A**) The schematic diagram of treatment. BUMPT cells were subjected to RLDC treatment for 96 h, followed by 7-day treatment of ABT-263 or vehicle (n= 8 experiments). (**B**) Representative images of the proliferation of post-RLDC surviving BUMPT cells at multiple time points during ABT-263 treatment. The boxed areas in the middle panels are enlarged for a close look at the formation of cell colonies outlined by dotted red curves. Scale bar: 1000 μm.

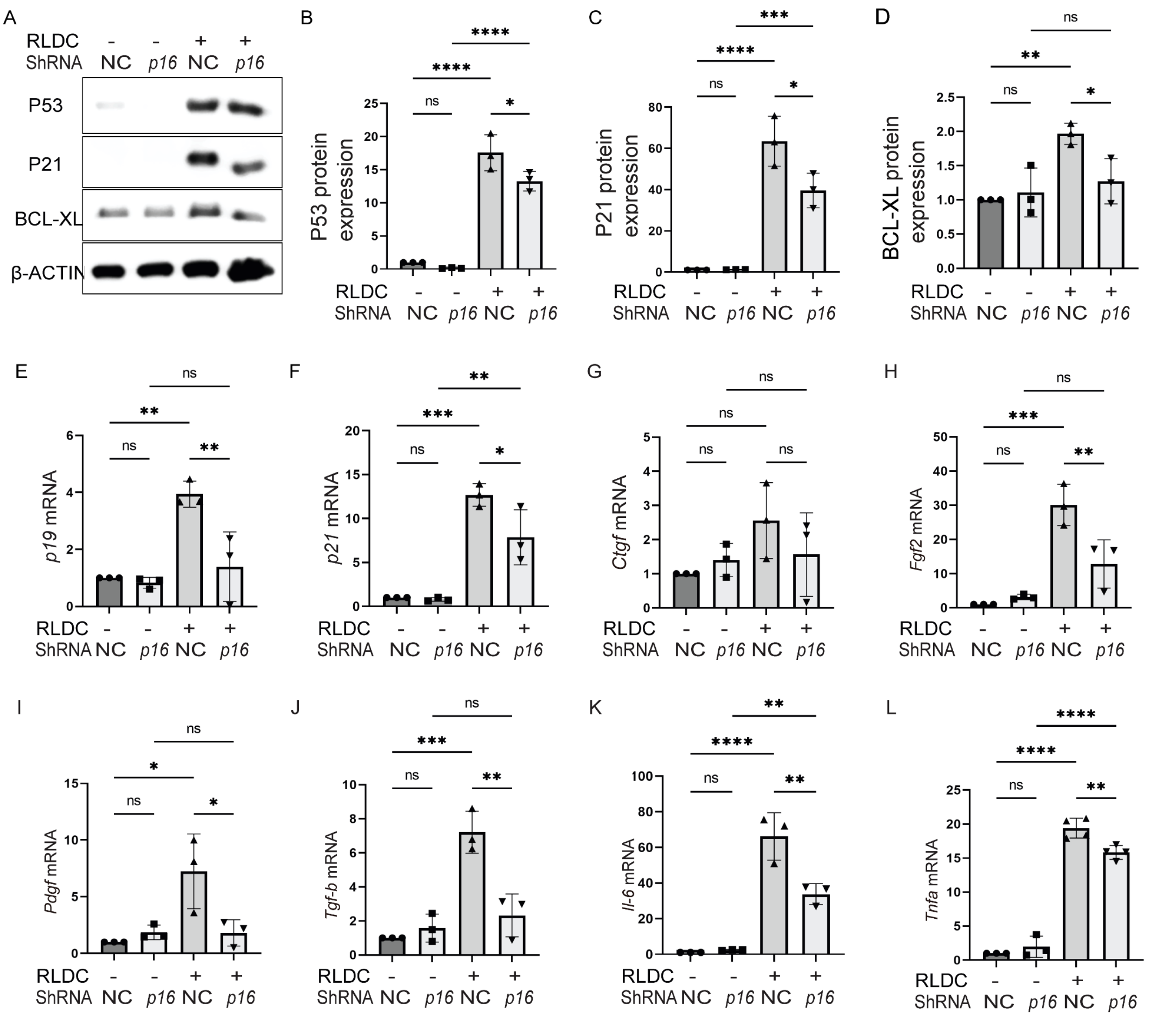

**Supplemental Figure 4. Knockdown of *p16* suppresses the induction of multiple senescence-regulating genes and SASP factors in RLDC-treated BUMPT cells.** BUMPT cells were transfected with *p16*-shRNA or negative control (NC) shRNA and then selected with puromycin for 2 weeks to establish stable cells for the subsequent RLDC treatment. (**A**) Immunoblot analysis for P53, P21 and BCL-XL. (**B-D**) Densitometry of P53, P21 and BCL-XL expression (n=3 experiments). (**E-L**) RT-PCR analysis of *p19*, *p21*, *Ctgf*, *Fgf2*, *Pdgfb*, *Tgfb*, *Il-6* and *Tnfa* mRNA expression (n=3 experiments). Quantitative data are presented as mean ± SD. Two-way ANOVA with multiple comparisons was used for statistics. *P < 0.05, **P < 0.01, ***P < 0.005, and ****P < 0.001.

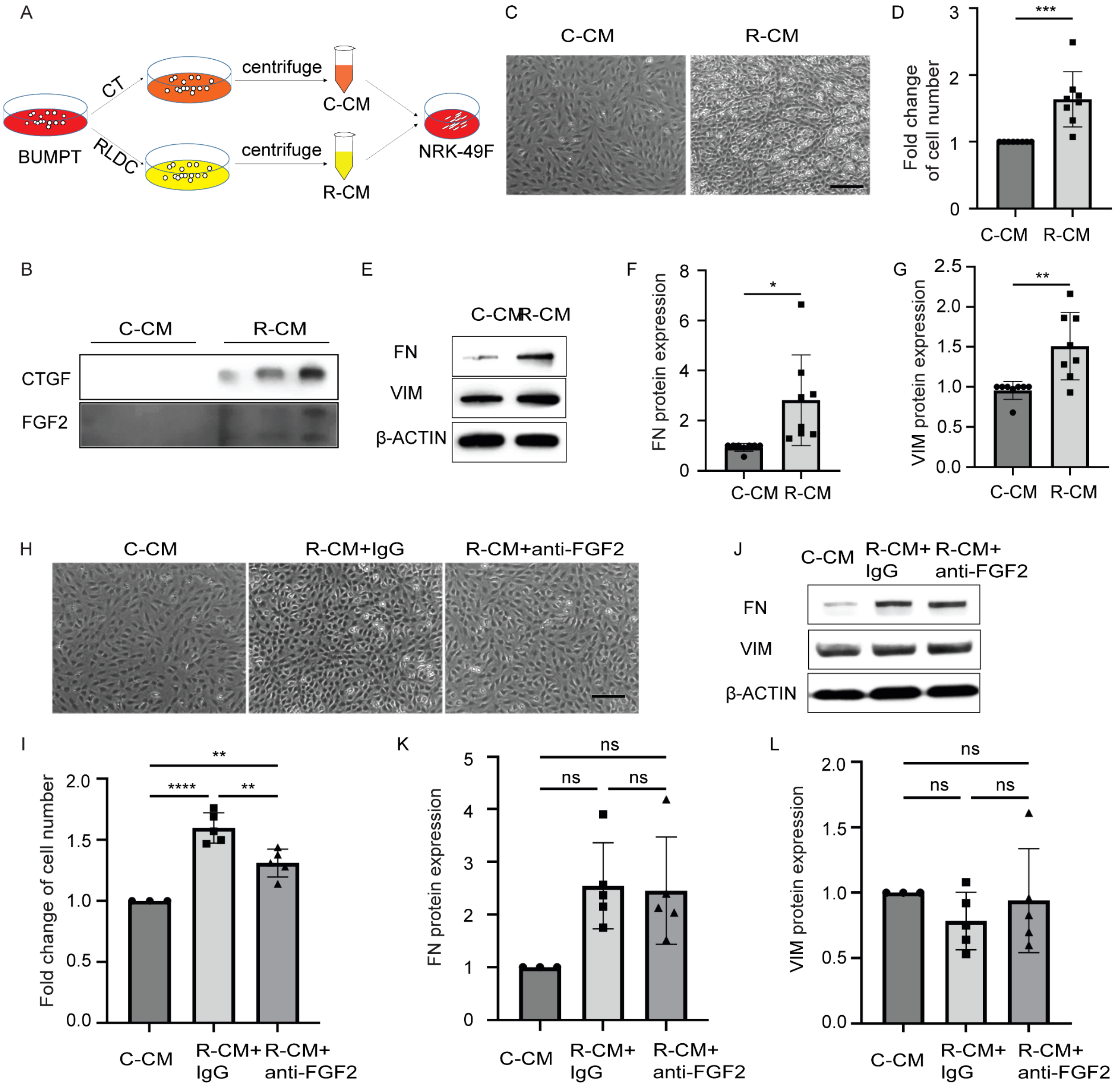


**Supplemental Figure5. RLDC-induced senescent tubular cells activates renal fibroblasts in a paracrine manner.**

(**A**) Schematic diagram of treatment. Conditioned medium was collected from either control BUMPT cells (C-CM) or post-RLDC BUMPT cells (R-CM). NRK-49F fibroblasts were incubated with serum-free medium containing C-CM or R-CM for 48 h (n=8 experiments). (**B**) Immunoblot analysis for secreted CTGF and FGF2 in CMs. (**C**) Cell morphology of NRK-49F fibroblasts. Scale bar: 400 μm. (**D**) Quantification of the fold changes of NRK-49F fibroblast cell numbers. (**E**) Immunoblot analysis for FN and VIM in NRK-49F fibroblasts. (**F** and **G**) Densitometry of FN and VIM expression. (**H-L**) Conditioned medium was collected from either control BUMPT cells (C-CM) or post-RLDC BUMPT cells (R-CM). NRK-49F fibroblasts were incubated with serum-free medium containing C-CM, R-CM with IgG (R-CM + IgG), or R-CM with FGF2 neutralizing antibody (R-CM + anti-FGF2) for 48 h (n=4 experiments). (**H**) Cell morphology of NRK-

49F fibroblasts. Scale bar: 400 μm. (**I**) Quantification of the fold changes of NRK-49F fibroblast cell number. (**J**) Immunoblot analysis for FN and VIM in NRK-49F fibroblasts. (**K** and **L**) Densitometry of FN and VIM expression. Quantitative data are presented as mean ± SD. For statistics, 2-tailed, unpaired t test was used for (**D**, **F** and **G**); one-way ANOVA with multiple comparisons was used for (**I**, **K** and **L**). *P < 0.05, **P < 0.01, ***P < 0.005, and ****P < 0.001.

| Gene | Forward primer | Reverse primer |
|---|---|---|
| *Mouse p16 mRNA* | 5′-CTC TGC TCT TGG GAT TGG C-3′ | 5′-GTG CGA TAT TTG CGT G-3′ |
| *Mouse p19 mRNA* | 5′-CAG TAG TAC CGG AGG CAT CT -3′ | 5′-TGA ACC GCT TTG GCA AGA-3′ |
| *Mouse p21 mRNA* | 5′-CTT GTC GTC TTG CAC T-3′ | 5′-AAT CTG CGC TTG GAG TAG-3′ |
| *Mouse Ctgf mRNA* | 5′-TTG ACA GGC TTG GCG ATT-3′ | 5′-GTT ACC AAT GAC AAT ACC TTC TGC-3′ |
| *Mouse Fgf2 mRNA* | 5′-GTC AAA CTA CAA CTC CAA GCA G -3′ | 5′-GAA ACA CTC TTC TGT AAC ACA CTT-3′ |
| *Mouse Pdgfb mRNA* | 5′- TTC CTC TCT GCT GCT ACC T-3′ | 5′-CAG CCC CAT CTT CAT CTA CG-3′ |
| *Mouse Tgfb mRNA* | 5′-GCG GAC TAC TAT GCT AAA GAG G-3′ | 5′- CCG AAT GTC TGA CGT ATT GAA GA-3′ |
| *Mouse Il-6 mRNA* | 5’-TCC TTA GCC ACT CCT TCT GT-3′ | 5′-AGC CAG AGT CCT TCA GAG -3′ |
| *Mouse β-actin mRNA* | 5′-GAT TAC TGC TCT GGC TCC TAG-3′, | 5′-GAC TCA TCG TAC TCC TGC TTG -3′ |

Supplemental Table 1. Summary of mRNA primers.
